# Antibodies as Biosensors’ Key Components: State-of-the-Art in Russia 2020–2021

**DOI:** 10.3390/s21227614

**Published:** 2021-11-16

**Authors:** Natalia Rudenko, Ksenia Fursova, Anna Shepelyakovskaya, Anna Karatovskaya, Fedor Brovko

**Affiliations:** Pushchino Branch, Shemyakin–Ovchinnikov Institute of Bioorganic Chemistry, Russian Academy of Sciences, 6 Prospekt Nauki, 142290 Pushchino, Russia; phursova_k@rambler.ru (K.F.); shepelyakovskaya@rambler.ru (A.S.); annakaratovskaya@mail.ru (A.K.); brovko@bibch.ru (F.B.)

**Keywords:** immunosensors, monoclonal antibodies, recombinant antibodies, protein scaffolds, lateral flow immunoassay, multiplex test systems, analytical microchips, nanosensors

## Abstract

The recognition of biomolecules is crucial in key areas such as the timely diagnosis of somatic and infectious diseases, food quality control, and environmental monitoring. This determines the need to develop highly sensitive display devices based on the achievements of modern science and technology, characterized by high selectivity, high speed, low cost, availability, and small size. Such requirements are met by biosensor systems—devices for reagent-free analysis of compounds that consist of a biologically sensitive element (receptor), a transducer, and a working solution. The diversity of biological material and methods for its immobilization on the surface or in the volume of the transducer and the use of nanotechnologies have led to the appearance of an avalanche-like number of different biosensors, which, depending on the type of biologically sensitive element, can be divided into three groups: enzyme, affinity, and cellular/tissue. Affinity biosensors are one of the rapidly developing areas in immunoassay, where the key point is to register the formation of an antigen–antibody complex. This review analyzes the latest work by Russian researchers concerning the production of molecules used in various immunoassay formats as well as new fundamental scientific data obtained as a result of their use.

## 1. Introduction

According to the current definition by the International Union of Pure and Applied Chemistry (IUPAC), a biosensor is a self-sustained integrated bioreceptor/transducer device intended to obtain selective quantitative or semi-quantitative analytical information using a biorecognition element [1]. A major part of developed devices are laboratory models and do not come into wide use. However, when speaking of affinity sensors, what is often meant is molecules or supramolecular structures specific to certain ligands and used in combination with special registration equipment. The main required parameters of affinity sensors are their detection rate, specificity, sensitivity portability, simplicity, ability to work with low sample volumes, and low cost [2]. The efficiency of affinity sensors depends on such factors as their ability to immobilize recognition elements (biological molecules) while preserving their natural activity, the accessibility of the recognition element for the relevant analyte in solution, and low, non-specific adsorption on a solid support. In this connection, high requirements are imposed on biosensors’ recognition molecules. The ligand should be highly specific, stable, and economical in production. For this reason, one of the main types of biosensors is biosensors based on antibodies.

This review is devoted to the research of Russian scientists in 2020–2021, in the field of the development and improvement of biosensors based on antibodies and their analogs. The reasons for using antibodies as key components of biosensors are discussed. Based on the examples of publications by Russian authors, it is shown that research is carried out on a broad front, affecting many areas of knowledge. Antibodies as sensors are used in fundamental research, diagnostic medicine, food analysis, and agrobiotechnology. The use of a variety of methods, both classical and modern, is demonstrated. The application and improvement of such methods as various variants of enzyme-linked immunosorbent assay (EIA), lateral flow immunoassay (LFIA), nucleic acid lateral flow immunoassay, and multiplex test systems based on analytical microchips, electrochemical, magnetic, and acoustic sensor systems are described.

For a long time, Russian science, including biotechnology, was far from the forefront of the world. Difficulties during perestroika (Russian for “restructuring”) had a significant role. However, in recent years, science has, for the first time, reached the rank of key national priorities. The field of science, to which this review is devoted, is developing at an accelerated pace, as evidenced by the growth of publication activity. For example, a search in PubMed (a database of medical and biological publications created by the US National Center for Biotechnology Information and the US National Library of Medicine) for the keyword “detection” reveals that the number of publications in 2020, in comparison with 2010, increased by 4.5 times. In Russia, technologies related to antibodies are widely developed and refined. This is contributed to by long-term traditions of research activities, backlogs/reserves in several key scientific areas, the drive for creativity and invention, and the existing large-scale network of scientific organizations. Russian science has become more international, and many scientific achievements are the result of international cooperation. The distinctive feature of Russian biotechnological science is the high rate of development, the creation of new directions practically from scratch, the openness for joint work with scientists from other countries, and the desire to integrate into the global scientific process. The purpose of this review is to summarize the achievements of Russian scientists in the development of immunosensors and introduce them to the target audience of the world’s scientific community, which will facilitate the search for scientific partners and the intensification of scientific cooperation.

## 2. Antibodies and Their Recombinant Analogs in Sensor Development

### 2.1. Monoclonal and Polyclonal Antibodies

Antibodies are the most widespread tools of modern biochemistry, cytology, and clinical medicine. Specific antigen–antibody interactions are the basis of diverse immunochemical methods. High-affinity antibodies, binding more antigens for a smaller period of time and forming more stable complexes, are preferable for immunochemical techniques. Antibodies, depending on their production technique, can be polyclonal or monoclonal (mAb) (Table 1 and Table 2). The activities of polyclonal antibodies are a combination of antigen-binding reactions of various antibodies. This makes the immunochemical method less concrete but more applicable for situations where the target is a group of structurally bound molecules. An inherent variability of polyclonal antibodies is explained by the different immune statuses of experimental animals. Whereas polyclonal antibodies are produced by numerous clones, mAbs are synthesized by one clone, which originates from one precursor plasma cell. mAbs are directed against only one particular epitope of an immunogenic substance and are identical in their structure, belong to the same class/isotype/allotype of immunoglobulins, have the same ligand-binding site structure, and possess the same specificity and affinity.

The first person in Russia to begin developing hybridoma technology as a method of producing monoclonal antibodies was Vasilov R.G. from the Shemyakin–Ovchinnikov Institute of Bioorganic Chemistry, Russian Academy of Sciences, Moscow. His first work studied an integral transmembrane protein, bacteriorhodopsin. Using mAbs as sensors, segments of the protein’s polypeptide chain expressed on the cell membrane surface were identified, which ultimately enabled, in combination with physico-chemical methods, the spatial structure of the enzyme to be established [3,4].

An advantage of mAbs is the possibility of standardization of antibody preparations. A concrete hybridoma cell line in mAb manufacture enables the stable production of antibodies and does not depend on the status of an animal, as is the case with polyclonal antibodies. The most important factor in determining a wide use of mAbs in various fields of science/medicine and branches of manufacture is, however, their fine specificity and directionality against a particular epitope. mAbs are used in diverse analytical studies. The development of conjugation technologies and the creation of various types of probes have revolutionized the field of immunodiagnostics. Depending on the tasks, many investigators and producers of diagnostic systems have successfully combined both monoclonal and polyclonal antibodies.

### 2.2. Development of Recombinant Technology in Antibody Design

**Figure 1:**
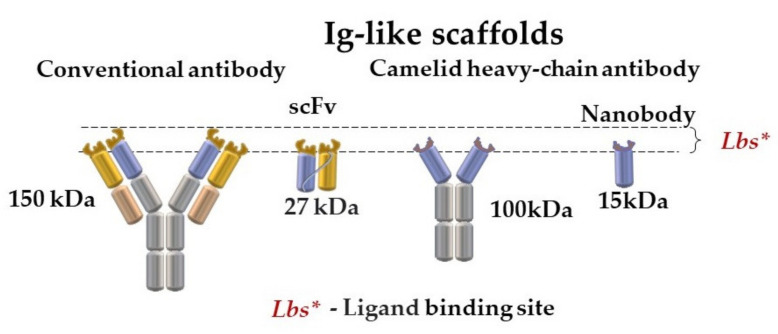
Immunoglobulin-like scaffolds.

With the development of display technology (phage, bacterial, yeast, ribosome, and mRNA) as a time-consuming and cost-effective speed and a cheaper method of antibody production in prokaryotic systems, various formats of recombinant antibodies with a number of necessary characteristics became accessible. Recombinant antibodies, such as Fab-fragments or a single-chain variable fragment antibody (scFv), compared to full-size natural antibodies, have a smaller molecular weight and increased molecular mobility, which provides them with more easy access to the antigen epitope (Figure 1, Table 1). This makes them an alternative to classical monoclonal antibodies [5].

Modern genetic engineering technologies and rational protein design open wide opportunities to change the properties of antibodies, creating more stable, affine, and less immunogenic molecules. Thus, the development of highly effective diagnostic systems requires increasing the affinity constant and an improvement in thermodynamic parameters and stability in storage. In [6], antibodies to botulinum neurotoxin type A underwent molecular evolution using random and site-directed mutagenesis. The newly produced antibodies had affinities exceeding the initial values by 35- and 81-fold. With these antibodies, the sensitivity of BoNT/A detection was 1 × 10^−13^ M [7].

The ability to work with recombinant antibodies as with recombinant proteins opens up a solution to several problems. In particular, immobilization of antibodies on the surface of a sensor is one of the key stages, which affects the detection limit, sensitivity, and general performance of the immunosensor. Unlike traditional antibodies, recombinant antibodies can be genetically engineered to self-assemble on biosensor surfaces at high density and optimized for correct orientation to enhance antigen-binding activity. Targeted immobilization of recombinant antibodies using genetically engineered tags, such as 6xHis-, myc-, Flag-, or ALFA-tag, makes it possible to maintain the efficiency of specific recognition of a required epitope by the antibody, in contrast with harder-to-control chemical crosslinking. Besides, a variant such as the introduction of histidines into the molecule of a recombinant antibody contributes to correct self-assembly on metal surfaces. Additionally, biosensor surface chemistry and physical and electronic properties can be modified to further increase immunoassay performance above and beyond that obtained by the use of traditional methods [8,9,10,11].

The prospects of applying antibody-improvement technologies can be significantly expanded by using rational approaches to the computer design of protein molecules. Thus, the authors of a recent work [12] used their earlier developed quantum mechanics/molecular mechanics (QM/MM) simulation methods for the maturation of the catalytic antibody A17 Ig-paraoxonase for organophosphorus toxins. The authors developed an algorithm based on a combination of density functional theory and funnel metadynamics. To enhance the nucleophilic attack of the target, they calculated an optimization of the amino acid design in the catalytic core. The developed algorithm enabled the production of molecules with expected properties. This approach can presumably be used to create artificial catalytic antibodies, as well as antibodies to cell surface receptors. It should, however, be taken into account that affinity-increasing targeted changes in the structure of antibodies can be accompanied by a violation of their specificity [13]. This emphasizes the importance of antibody-validation procedures after the improvement stage [14].

#### 2.2.1. Recombinant Antibodies in Detection Systems

Of special interest both in Russia and all over the world have been, in recent years, single-domain antibodies (nanobodies) (Figure 1) and their derivatives. Nanobodies consist of a variable domain of only heavy-chain (VhH) camelid (Llama and Camel) immunoglobulin. Several features such as small molecular weights (12–15 kDa), stability, affinity, and recognition of structural epitopes enable high hopes to be laid on this molecule both in diagnosis and in immunotherapy [15,16,17,18,19]. A detailed description of approaches to the production of bispecific antibodies using single-domain antibodies from *Camelidae* is given in [20]. Adaptive single-domain antibodies are used for molecular visualization and proteomic immunoassay [21], the development of novel immunosorbents and detection methods [5], and cell and tissue target recognition. Thus, recent work in this field has dealt with developing a microcantilever immunosensor using an anti-carcinoembryonic antigen nanobody VhH [16]. In this work, a nanobody was covalently immobilized onto the surface of the sensor and worked as a receptor molecule for catching antigens from solution. The detection limit of antigens in developed construction is 0.03 ng/mL. The efficiency of the method was ensured by the small size of the nanobody and, accordingly, by the small distances between antigen-binding regions and sensor surfaces, which significantly elevated the generation and transmission of surface stress.

Recombinant antibody technologies are successfully used in the development of test systems for the detection of toxins and venoms. The diagnosis of arachnid bites remains rather difficult as of today. This is due to several restrictions in the development of the assay: (i) the complexity of reliable identification of the nature of a venom, (ii) the speed of assay execution due to the rapid propagation of venom in tissues, and (iii) high sensitivity of the assay, which is due to very low venom doses in bites and their rapid distribution in the blood [22]. A recently published work by a team of researchers from Denmark, Russia, and the United Kingdom [23] describes the production of α-latrotoxin recombinant human monoclonal immunoglobulin G antibody using a phage display library with primary scFv selection and its conversion to IgG. It was expressed in a HEK 293 cell line. The antibody possessed both detecting and neutralizing properties. Although it was produced against α-latrotoxin from *Latrodectus tredecimguttatus*, the authors believe that the antibody can also be used to detect other α-latrotoxins and is a promising agent for diagnosis and therapy.

To date, numerous unique proteins involved in various bioluminescence reactions have been discovered owing to molecular-level research into organisms from terrestrial or aquatic ecosystems. Among these proteins are luciferases and Ca^2+^-regulated photoproteins, used for a long time as reporter molecules in in vitro and in vivo assays. Herewith, bioluminescent proteins act as a constituent part of the bifunctional sensor molecule in combination with the antigen-recognizing module, e.g., an antibody. The production of bifunctional molecules by methods of genetic engineering has several advantages compared to molecules produced by chemical-crosslinking agents. These are primarily their invariable ratio of the recognition and reporter components and their constant orientation with respect to each other. A test system based on the sandwich-type bioluminescent immunoassay has been developed [24]. This work constructed hybrid bifunctional proteins consisting of the recognition domain—a murine single-chain variable fragment mini-antibody (scFv 14D5a) specific to glycoprotein E of the tick-borne encephalitis virus. The MLuc7 isoform of 16.5 kDa luciferase from *Metridia longa* was used as the reporter domain. It is, in the opinion of the authors, exactly the small size of the molecule that can give sizeable advantages compared to other bioluminescent proteins, such as *Renilla* or firefly luciferase, because the probability of steric hindrances decreases with a smaller size of sensor molecule reporter module. Besides, the authors point out the possibility of modifying both the N- and C-termini of the molecule, which makes the system readily adaptable for various assay formats. It should, however, be noted that Mluc7 is the most active at a temperature of 12–15 °C, and besides, it is difficult to accumulate it in a prokaryotic expression system. Site-directed mutagenesis of reporter molecules and the use of other expression systems can increase the potential of bioluminescent detection systems even more. Thus, expression in insect cells enables a high yield of the hybrid protein. The detection limit of the tick-borne encephalitis virus using a scFv–MLuc7 construct exceeds, by 26-fold, the one using the colorimetric method. The suitability of the construct was tested on biomaterial from 144 ticks from natural populations.

#### 2.2.2. Phage–Antibody Conjugates in Detection Systems

**Figure 2:**
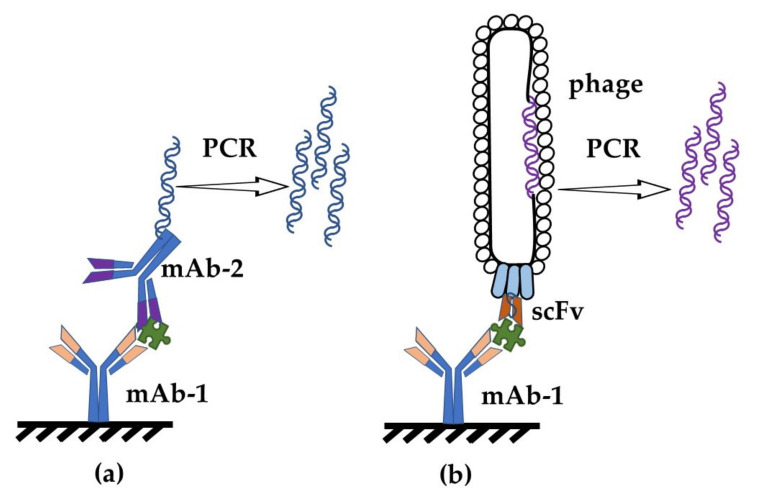
Schematic representation of immuno-PCR (**a**) and phage-display-mediated immuno-PCR (**b**) in antigen detection.

Despite the active development of instrumental analysis methods, one of the most widespread laboratory techniques for detecting agents of various natures in biological mixtures is the immuno-PCR [25]. This is a sensitive method based on using the DNA matrix bound to the antibody either covalently or via the biotin–streptavidin complex. The choice of the antibody/DNA conjugation strategy is of critical importance because it determines both the efficiency of the conjugation reaction itself and the activity of the obtained product and, as a consequence, the sensitivity of the assay (Figure 2a). For this reason, various conjugation techniques have been developed and optimized [26,27]. Alternatively, phage-display-mediated, real-time immuno-PCR enables the conjugation problem to be worked around. This method is based on using a phage-displayed antibody as a detecting agent—usually, these are recombinant antibodies (scFv, VhH) produced using the phage-display technique [28,29,30,31]. The antibody exposed on a phage particle acts as an agent binding to the antigen; the phage DNA, with the incorporated gene of the antibody, serves as a matrix for the PCR (Figure 2b). The procedure of producing this biological conjugate of the phage and the antibody is rather simple and requires no complex means of antibody purification. This method was used to detect staphylococcal enterotoxin A (SEA) in milk [28]. The method was developed in a sandwich version, where monoclonal antibodies were used for trapping, and detecting antibodies were phages exposing anti-SFA scF for detection. The detection level was 100 pg/mL, which is much lower than the level of the toxic and allergic doses for humans.

Another interesting work in phage conjugates is the Cytometry Cell-Labeling Operable Phage Screening (CyCLOPS) platform [32]. This development is based on using the phage-display technology using the fADL-1e-phage vector for high-performance screening of B- and T-cell-surface antigen ligands. With the help of this vector, phages efficiently expose the specified peptides on their surface, which are used in the selection procedure together with a cytofluorimetric analyzer. The authors also report that the CyCLOPS can be efficiently used for the screening of antigen-specific lymphocytes and HLA class II peptide epitope screening. The system can be successfully used to search for receptor ligands in either cell-based or surface-immobilized assays.

#### 2.2.3. Non-Immunoglobulin Alternative Protein Scaffolds in Detection Systems

Great attention has been paid worldwide to alternative antigen-binding molecules of a non-immunoglobulin nature, scaffold proteins, as a promising biotechnological tool (Figure 1). This actively expanding group includes adnectins, affibodies, anticalins, bicyclic peptides, DARPins, fynomers, and Kunitz domains [33,34,35]. These proteins have several advantages, which makes them a successful alternative to antibodies (Table 1 and Table 2).

**Figure 3:**
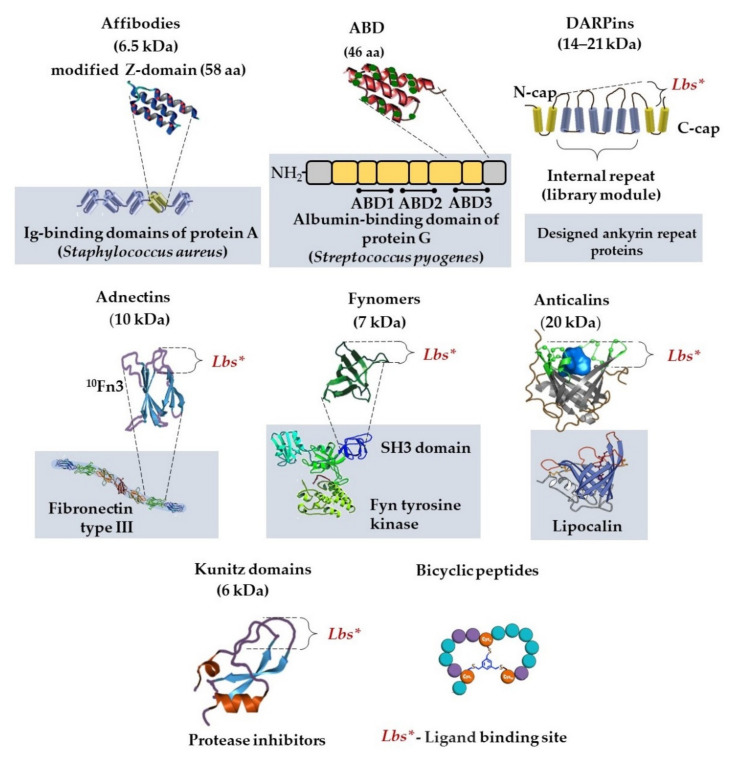
Non-immunoglobulin alternative protein scaffolds.

Protein scaffolds are much smaller than a full-size antibody, and their molecular weights are from 2 to up to 20 kDa, versus 150 kDa for a full-size monomer antibody (Figure 3). 

Alternative protein scaffolds are characterized by high stability [36], low immunogenicity, and high yield with *Escherichia coli* expression (much higher than for recombinant antibodies). Based on those, bi-, tri-, and tetra-specific compounds are constructed for simultaneous binding of several epitopes from different targets [37,38].

The largest number of publications in Russia on scaffold proteins are regarding designed ankyrin repeat proteins (DARPins). Owing to their unique properties, DARPins are used for diagnosis and therapy: cancer diagnosis and therapy [39,40,41,42,43], DARPin-based tumor-targeting toxins [44,45,46], nanoparticle delivery, and designing oncolytic viruses and chimeric antigen receptors. Several properties and application of analyte-binding scaffolds are shown in Table 2.

Another variant of a scaffold protein is an affibody. The affibody molecule is based on a Z domain of *Staphylococcus aureus* surface protein A. It is a 58 amino acid modified peptide that binds to the Fc domain of antibodies. Randomization of 13 amino acids on two out of three helices of the Z domain enables display libraries to be constructed, from which molecules with specified properties can subsequently be taken. Due to their small size, affibodies gain advantages for diagnostic imaging [35].

A recent work [47] has shown that affibody ZHER2:342 specifically binds to cells of the human breast adenocarcinoma cell line SK-BR-3 with overexpression of the HER2 receptor. Authors developed polymer-nanoparticle-bearing affibody ZHER2:342, which produced reactive oxygen species when filled with a fluorescent xanthene dye, Rose Bengal. This affibody construct has a double effect on diagnostic (fluorescent) and cytotoxic effects simultaneously. In addition, in the previous work, the authors compared various types of HER2-targeting scaffolds in nanoparticles. They synthesized nanoparticles with a set of properties: magnetic, fluorescent, and directed toward the HER2 oncomarker on cancer cells. Magnetic particles were covalently modified: full-size IgG (150 kDa), DARPin_G3 (14 kDa), and affibody ZHER2:342 (8 kDa). The smallest HER2-recognizing polypeptide affibody ZHER2:342 was shown to be more effective in specificity and selectivity in nanoparticle-mediated cell labeling [43].

In [48], the use of a new transporter for the delivery of affibody Z1907 into tumor cells is described. The authors developed a new modular nanotransporter that delivers ligands, providing a cytotoxic effect to the nucleus of a target. 

Miniaturization of specific-antigen-recognizing molecules as specific molecular probes is of important significance for the visualization of metastasizing tumors. A rather promising platform in this respect is the Albumin-binding domain (ABD) Derived Affinity ProTein (ADAPT), the basis of which is the albumin-binding domain of streptococcal protein G. The small size of the molecule (46–59 amino acid residues) and affinity at the level of nanomolar values gives grounds to believe that this type of molecule can be successfully used as an imaging agent. Thus, it has been shown in the course of preclinical and the first stage of clinical tests that HER2-specific, radioactively labeled ADAPT6 demonstrates a high-affinity recognition of HER2 in tumors. Its use as a complex with 99mTc-ADAPT6 makes it a promising agent for target recognition in radionuclide molecular imaging [49].

## 3. Monoclonal Antibodies as Tools to Study Biological Molecules and Intermolecular Interactions

In modern Russia, the tradition of using mAbs to study biologically active compounds continues. Owing to their high specificities, antibodies are extensively used as molecular sensors in studies of the structure, function, and localization of biological molecules.

The following are examples of how authors have successfully used monoclonal antibodies to study biological macromolecules. YB-1 protein plays an important role in the development and regulation of inborn immunity reactions. Using fluorescently labeled antibodies, the intracellular localization of YB-1 protein was determined. With the application of phage-display technology, the peptide interacting with YB-1 protein was found. Based on monoclonal antibodies MAYB-1-2 and a peptide as part of the bacteriophage structural protein, a test system for the quantitative determination of YB-1 protein in blood serum was developed [50]. Using the sandwich immunoenzyme analysis developed based on mAbs, the intra- and extracellular localizations of the molecular forms of highly homologous lytic endopeptidases AlpA and AlpB from *Lysobacter* sp. XL1 were determined. Despite the general principle of synthesis and homology, the enzymes AlpA and AlpB differ in how they are secreted into the environment [51]. The mAbs described in [52] bind to two different epitopes of G protein of the respiratory syncytial virus, possess a virus-neutralizing activity, demonstrate an additive effect in interaction, detect the viral antigen in infected cell cultures, and can be used to reveal the virus in clinical material by immunofluorescence assay and ELISA. Studies using mAbs have shown the significance of the C-terminal domain of hemolysin II for the hemolytic activity of *Bacillus cereus* (which causes infections in persons with weakened immunity and is one of the widespread causes of intra-hospital infections) [53], as well as its ability to bind to cell membranes [54] and be oligomerized in the presence of cell membranes [55].

Of particular relevance at present is work aimed at the development of SARS-CoV-2 detection and therapy systems. It has been found that the diagnostically significant and, at the same time, major protective antigen of the virus is the receptor-binding domain of the SARS-CoV-2 spike protein S. Production of murine mAbs against this antigen has been described, and their ability has been shown to specifically reveal native and denatured S proteins in immunoblotting and immunoenzyme assays, as well as in samples of tissues from patients in immunocytochemical and immunohistological studies. A distinctive feature of the produced antibodies is their ability to efficiently block SARS-CoV-2 infection in vitro. Based on the information on the primary structure of these mAbs, it is expected in the future that immunotherapeutic drugs will be developed by genetic engineering methods. Owing to the high mutation variability of SARS-CoV-2 strains, work on the production of mAbs against various variants of proteins of this virus are as relevant as ever and are being actively continued [56].

Based on the amino acid sequence data for two mAbs against morphine (3K11 and 6G1), by docking simulations, the 3D models of the complexes of morphine and variable regions of the Fab fragments of these antibodies have been obtained. Analysis of the obtained 3D models enabled differences to be revealed in the positions of the ligand (morphine) relative to the antigen-binding sites of these antibodies. This correlated with the data on the differences in the epitope specificity of these antibodies obtained experimentally by surface plasmon resonance and an immunoenzyme assay. Subsequently, accounting for the obtained data, it is possible to construct humanized anti-morphine antibodies that can be used for therapeutic purposes [57].

mAbs against the FLAG peptide sequence (DYKDDDDK) (F1804, Sigma-Aldrich, St. Louis, MO, USA) were used to investigate the molecular mechanisms of eukaryotic transcription activation, including the coordination of several hundred proteins in a limited space, as exemplified by the 20-hydroxyecdysone (20E)-dependent *Drosophila melanogaster* developmental genes. The main molecular sensor of this system is the ultraspiracle protein/ecdysone receptor (USP/EcR) heterodimer; EcR contains FLAG. The heterodimer is bound to regulatory DNA regions and, depending on the transcription status, to coactivators or corepressors. For biotin labeling of proteins enclosing the heterodimer, the biotinylation enzymes (BioID2 (biotin protein ligase) and APEX2 (peroxidase)) were fused to EcR or Usp. Detection of biotin, dependent on proximity, revealed earlier-undescribed participants of the ecdysone response in the drosophila. Contact interactions of the EcR/Usp heterodimer with enclosing biotinylated proteins were determined. The association of EcR/Usp with CP190 protein—a well-described cofactor of *Drosophila* insulators—was investigated. The obtained results support the concept that CP190 is important for the stabilization of chromatin-specific loops for the correct activation of the transcription of genes regulated by the 20E hormone [58].

Interactions between complementary (or heterologous) mAbs and two types of lipopolysaccharides from the genus *Yersinia* were investigated by force spectroscopy. Determining the strength of intermolecular interaction between the antigen and the mAb can be an efficient approach to assessing the mAb affinity required to develop test systems or immunosensors; it is also useful for understanding the interaction between the lipopolysaccharide and the mAb. Experimental data for the optical trapping on a sensitized polystyrene microsphere-sensitized glass substrate model system at an approach/retraction in the vertical plane have been obtained [59].

One study [60] describes the production of high-affinity murine mAbs against synthetic nona-β-(1→3)-d-glucoside, a constituent part of the cell wall of many pathogenic fungi, such as yeasts, bacterial species from the genera *Aspergillus*, *Candida*, *Penicillium,* and *Saccharomyces cerevisiae*. Detection and quantitative evaluation of this polysaccharide is an important challenge for clinical diagnosis, food control, and ecology monitoring. In this study, the authors showed the potential of these mAbs to localize β-(1→3)-d-glucan in the fungal cell wall, inhibiting fungal growth, and in the combinatorial antifungal therapy, the described properties of mAbs assume a high probability of use in the development of diagnostic kits.

## 4. Production of Monoclonal Antibodies and Immunodiagnostic Kits in Russia

To date, the production of monoclonal antibodies has become a routine task throughout the world. Researchers waste no time or effort on proven methods. Russia is not lagging behind, either; companies manufacturing immunochemical reagents are actively developing in that country. Successfully operating companies include Generium (Moscow, https://www.generium.ru/; 28 September 2021), Vector-Best (Novosibirsk, https://vector-best.ru/en/; 28 September 2021), Diagnosticheskie Sistemy (Nizhny Novgorod, http://www.npods.ru/; 28 September 2021), *Vetbiokhim* (Moscow, http://vetbio.ru/; 28 September 2021), Bialexa (Moscow, http://www.bialexa.ru/technical-support/faq/; 28 September 2021), and others. Russian companies produce a wide spectrum of monoclonal antibodies specific to various protein markers, antigens, and antibody derivatives, as well as kits for the diagnosis of HIV, viral hepatitis, sexually transmitted infections, herpes virus infections, tick-borne and zoonotic diseases, helminthiasis, tuberculosis, anemia, gastrointestinal and cardiovascular diseases, kits for the detection of cancer markers and acute-phase proteins and for the quantitative determination of hormones and cytokines, etc. Companies actively cooperate with scientists who use their products as research tools in various fields of theoretical and applied biochemistry.

In 2020–2021, many publications appeared that demonstrate examples of the successful use of domestic manufacturers’ products. For instance, kits manufactured by Vector-Best (Novosibirsk, Russia) for immunoenzyme assays (D-5501 SARS-CoV-2-IgG-EIA-BEST and D-5502 SARS-CoV-2-IgM-EIA-BEST) were used in research to assess the spread of seropositivity among symptomatic individuals and those with a high risk of occupational exposure to SARS-CoV-2 [61]. According to the data obtained, only 47% of symptomatic persons and 17% of those with a high risk of occupational exposure are seropositive to SARS-CoV-2.

The Vector-Best IgG-measles test system (Russia) was used in a study [62] to investigate the level of immunity to the measles virus in women in labor and staff of maternity hospitals in one medical institution. It was shown that the studied contingent is a risk group for measles due to the high proportion of seronegative individuals among women of childbearing age (both maternity hospitals’ personnel and women in labor).

An integral part of the development of diagnostic drugs is the evaluation of the effectiveness of all technological stages. One of the recent examples is [63], in which commercial fluorescent immunoglobulins were characterized by spectrophotometric analysis. The work improved the fluorescent immunodiagnostic kits for assaying particularly dangerous infections (tularemia, plague (Mikrob Russian Antiplague Research Institute)).

Reagents manufactured by the Russian companies Diagnosticheskie Sistemy and Sorbent-servis have been successfully used for the development of the first Russian diagnostic kit, Blot-HEV, intended for the detection of IgG antibodies against particular proteins of hepatitis E virus (HEV) in human blood serum using the linear immunoenzyme assay, in which recombinant antigens belonging to HEV, as well as control antigens, were immobilized on a nitrocellulose membrane [64].

## 5. Immunodiagnostic Systems Based on Nanomaterials 

**Figure 4:**
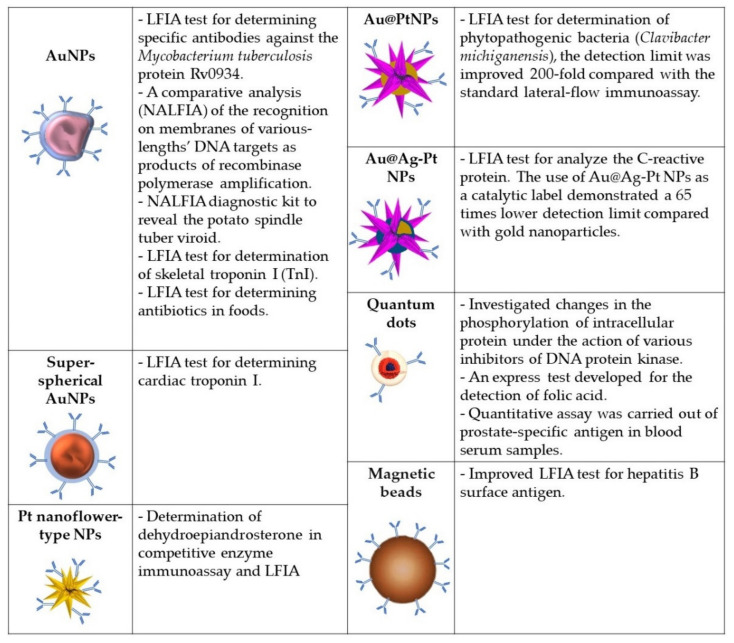
Useful modifications of nanoparticles for improvement of the analyte detection described in this review: AuNPs [65,66,67,68,69,70,71,72,73], Superspherical AuNPs [74], Pt-nanoflower-type NPs [75], Au@PTNps [76], Au@Ag-PtNPs [77], quantum dots [78,79,80], magnetic beads [81].

The distinctive features of some nanomaterials, such as tunable optical properties, the ability to self-assemble, and catalytic and ferromagnetic activity, make them attractive for use in the latest immunoassay systems. For about half a century, gold nanoparticles (NPs) have been used as a marker in immunochemistry. Due to the simplicity and speed of application, immunochromatographic test systems have now become very popular and are widely used. Figure 4 summarizes the application data and the various types of nanoparticle modifications described below.

### 5.1. Immunochromatographic Test Systems (LFIA)

Immunochromatographic test systems are widespread worldwide owing to the simplicity of their use and visualization of the results [82,83]; their mass production has been established. More often, immunochromatography is used to determine the content of specific antibodies in studied biological fluids. Antibodies that appear in human and animal bodies in response to an encounter with a foreign antigen can be of diagnostic value under various conditions; therefore, they can be not only a component of the detection system but also its analyte.

The desire to achieve perfection moves Russian scientists towards further improvement of this technology; new tests for the diagnosis of infections and pathologies are being actively developed.

In [65], mathematical methods were applied to refine the LFIA. The authors believe that their developments will be of use to avoid false-positive results, decrease the detection limits, and optimize conditions in transition to other analytes. They mathematically investigated the formation of analyte-detecting agent complexes and analyzed factors affecting the system’s detection limits; recommendations were proposed to reduce them. The mathematical conclusions were confirmed by determining specific antibodies against the *Mycobacterium tuberculosis* protein Rv0934 (mAbs HTM81; Centre for Molecular Diagnostics and Therapy (Moscow)).

In [84], the Alarm-LFIA test was developed for rapid and simultaneous assaying of five potato viruses (viruses X, M, S, Y, and potato leaf roll virus) with detection limits from 10 to up to 30 ng/mL in plant extracts. The “alarm” prefix signifies that the detection of at least one of the analytes is indicative of infection. To increase the sensitivity of the Alarm-LFIA, its constituent components are being developed and improved [85,86]. In [74], the replacement of commonly used quasispherical-citrate-capped gold nanoparticles by superspherical gold nanoparticles using the Turkevich–Frens technique was demonstrated to lead to a significant increase in the sensitivity of the method. Cardiac troponin I was used as target and anti-troponin mAbs, manufactured by Bialexa, Moscow, were used for detection.

The enzyme-like activity of metal nanoparticles (nanozymes) has made it possible to develop combined immunodetection methods that combine the convenience and speed of LFIA with the accuracy and high sensitivity of enzyme immunoassay detection methods. In [75], it was demonstrated that using antibody conjugates with platinum nanoflower-type nanoparticles as detection agents in competitive enzyme immunoassay and LFIA provides good selectivity and high sensitivity in the determination of dehydroepiandrosterone, a multifunctional steroid hormone. The high stability of nanozymes compared to conventional enzyme preparations determines the relevance of more detailed studies of the properties of nanoparticles and the development of diagnostic systems based on them.

The authors of [76] investigated the effect of the composition of Au@Pt nanoparticles, their morphology, and peroxidase-like activity on the detection limit of the lateral flow immunoassay. Fourteen types of nanoparticles were synthesized by varying the concentration of Pt^4+^ (20–2000 μM) using gold nanoparticles (diameter, 20.0 ± 2.6 nm) as a seed and ascorbic acid as a reducing agent. Nanoparticles of Au@Pt and gold nanoparticles were conjugated with polyclonal antibodies specific to the target analyte, a widespread and hazardous species of phytopathogenic bacteria (*Clavibacter michiganensis*) affecting potatoes and other cultures by causing tuber ring rot in the field and storage. An increase in the concentration of Pt^4+^ was found to lead to an increase in the peroxidase activity and a decrease in the analyte detection limit. The greatest sensitivity of the method was achieved for Au@Pt nanoparticles synthesized at a Pt^4+^ concentration of 1000–1200 μM. The detection limit for bacteria in a plant extract was improved 200-fold compared to the standard lateral-flow immunoassay.

Another approach providing enhanced catalytic activity of nanozymes was to create nanoparticles with an effective dispersion of Pt atoms on their surface owing to a modified synthesis technology [77]. Namely, the synthesis was conducted in three stages: synthesis of gold nanoparticles and the building of a layer of silver on them, followed by the galvanic substitution of PtCl_6_^2−^ for silver. As a result, trimetallic nanoparticles of Au@Ag-Pt were obtained with uniformly applied catalytic centers and high efficiency of Pt use. The produced nanoparticles were conjugated with mAbs (HyTest, www.hytest.fi) and used as colorimetric and catalytic labels in the lateral flow immunoassay to analyze the C-reactive protein inflammation biomarker. The use of Au@Ag-Pt nanoparticles as a catalytic label demonstrated a 65-times lower detection limit of C-reactive protein in serum (15 pg/mL) compared to gold nanoparticles.

### 5.2. Nucleic Acid Lateral Flow Immunoassay

A new promising application of lateral flow tests is the detection of genetic material. The method is called nucleic acid lateral flow immunoassay (NALFIA). Combining isothermal nucleic acid amplification and lateral flow immunoassay provides a highly sensitive non-laboratory (point-of-care or field) detection of an analyte of interest. However, to obtain correct results and maximally achievable sensitivity parameters, it is important to choose the size of the detected amplicons. A comparative analysis of the recognition on membranes of various-length DNA targets as products of recombinase polymerase amplification was carried out in [66]. That work used mAbs against fluorescein (clone 2A3c, Bialexa (Russia)). For visualization, DNA targets were functionalized with biotin and fluorescein, and gold nanoparticles were conjugated with antibodies against fluorescein and streptavidin. The detection limit was 70 pM for a target of a length of 150 bp [87]. Murine mAbs specific to fluorescein (anti-FAM) (clone 2A3c, Bialexa, Russia) were used when developing a diagnostic kit based on the NALFIA principle to reveal the potato spindle tuber viroid [67]. In [81], a test for hepatitis B surface antigens was developed, in which the LFIA was improved by the electronic registration of superparamagnetic nanolabels.

### 5.3. Optical Methods of Immunodetection Based on Nanomaterials

The use of traditional organic fluorophores has several limitations. They cannot be used to conduct long-time studies, as most organic fluorophores lose color with time. A good alternative to traditional organic fluorophores is using various recently developed nanomaterials, such as semiconductor quantum dots, porous silicon semiconductor nanoparticles, carbon dots, gold nanoclusters, etc. [88,89,90,91,92,93].

Quantum dots as a source of fluorescent signals are also used in immunoassay formats on microchips. Thus, the authors of [78] investigated changes in the phosphorylation of intracellular protein under the action of various inhibitors of DNA protein kinase in glial cell lines. In this work, analyte detection was based on using the sandwich approach with streptavidin–biotin to assess and monitor the fluorescence signal instead of direct labeling of samples, which helped improve the reproducibility of the results and the sensitivity of the microarrays. The use of quantum dots led to a significant improvement in sensitivity and specificity compared to organic fluorophores.

Rather attractive is the use of bright water-stable quantum dots combined with an immunoassay format as popular as the LFIA. As a competitive solid-phase enzyme immunoassay, the LFIA is frequently used to develop systems of detection of various low-molecular-weight analytes. For instance, in rather high demand are highly sensitive and inexpensive test systems for assaying the quality of foodstuffs, which would make it possible to assess the content of biologically active substances within minutes. An express test developed for the detection of folic acid with a visual assessment of the results using quantum dots is described in [79]. Moreover, the authors gave up on Cd-based quantum dots, which are the most popular for similar purposes owing to their potential toxicity, and used hydrophilic photostable AgInS/ZnS nanocrystals. These nanocrystals were conjugated with mAbs against folic acid produced by Bialexa. The prepared AgInS/ZnS-based LFIA presented a high precision rate of 97.2%. A linear range was obtained for the folic acid concentration range of 0.9–6.7 μg/mL. 

Another immunoassay format, which requires bright and stable fluorophores, is flow cytofluorometry. At present, the “gold standard” for serological detection of particular analytes is EIA; however, for many biomarkers, multiplex test systems for flow cytofluorometry that are not inferior by sensitivity and specificity have already been developed and are being used. Using mAbs conjugated with quantum dots, a quantitative assay was carried out on prostate-specific antigens in blood serum samples from prostate cancer patients and healthy subjects. This method can be readily adapted to reveal other diagnostically significant disease markers [80].

## 6. Practical Topical Applications of Modern Immunosensor Systems

### 6.1. Screening of SARS-CoV-2 Antibodies

Russia is among the countries affected by SARS-CoV-2, and its health authorities have mobilized significant efforts and resources to fight the disease. The pandemic caused by SARS-CoV-2 has not, unfortunately, stopped; work on the development of detecting test systems to enable the progression of the population immune response to be monitored continues intensively. Detection of antibodies against SARS-CoV-2 (a severe acute respiratory syndrome) plays an important role in the fight against the pandemic.

Linear peptide epitopes of about 15 aa of the SARS-CoV-2 spike protein were selected using antigenicity algorithms and synthesized with an additional terminal cysteine residue for their bioconjugation. Constructs were synthesized in which the peptide (F, function) was attached to a negatively charged hydrophilic spacer (S) linked to a dioleoylphosphatidyl ethanolamine residue to create a function–spacer–lipid construct (FSL). These FSLs were easily and controllably incorporated into erythrocytes for serologic testing (kodecytes—altered erythrocytes—were formed) or into a lipid bilayer deposited on a polystyrene microplate for use in enzyme immunoassays. For comparison, the same peptides were condensed with polyacrylamide. It turned out that kodecytes with an embedded peptide showed the best specificity and sensitivity [94].

In continuation of the previous work, the possibility of using erythrocytes (C-19 kodecytes) modified using Kode-technology function–spacer–lipid constructs and carrying short SARS-CoV-2 peptides was assessed to develop an assay compatible with the existing routine serological platforms. It has been shown that C19-kodecytes are viable for use as erythrocytes of a serological reagent to detect SARS-CoV-2 antibodies using standard equipment for screening antibodies in the blood [95].

For immunological screening of antibodies to SARS-CoV-2, a platform was developed [96] using recombinant SARS-CoV-2 antigens: a spike protein (S-protein), its receptor-binding domain (RBD), its subdomains (RBD, -SD1, and NTD), and a nucleocapsid protein (N-protein). The system included both linear and conformational epitopes. Humoral reactions of adaptive immunity in cohorts of Russian-population patients in response to COVID-19 infection were characterized. The advantage of class A immunoglobulins as an early immunological criterion for the development of the disease was reliably established. The spectrum of specificity of immunoglobulins caused by SARS-CoV-2 in each patient depended on the time after infection and varied in the series of immunoglobulins M, A, and G from narrow to wide. Uneven induction of immunoglobulin subclasses was also shown; it depended on the nature of the antigen. It has been shown that the N-protein induces immunoglobulins G1–G4 and A1–A2 in equal proportions, whereas G1, G3, and A1 are the main subclasses of the immune response to the S-antigen.

The epitope-specific immunological landscape of the breast milk of women recovered from COVID-19 and who were cured of COVID-19 in different trimesters of pregnancy was carried out by comprehensive screening [97]. An EIA diagnostic kit based on recombinant antigens and fragments of the SARS-CoV-2 virus protein was developed. The analysis showed an increase in samples with a conformation-dependent sIgA to the receptor-binding domain, i.e., neutralizing antibodies. Breast milk can be a potential means of protection against COVID-19 for infants, giving them passive immunity.

### 6.2. Immunochemical Detection Systems for Assaying Foodstuffs

In Russia, as in other countries of the world, the importance of food quality is understood. Access to a sufficient number of safe and nutritious foodstuffs is an essential factor for maintaining human life and promoting human health. Unsafe foods containing pathogenic infectious agents or harmful chemicals threaten human health and cause economic damage associated with decreased labor productivity and increased medical costs.

A group of researchers from the A.N. Bach Institute of Biochemistry, Research Centre of Biotechnology, Russian Academy of Sciences, has been successfully developing immunochromatographic test systems to analyze foodstuffs. For the analysis of the quality of meat products, the following were developed: a lateral-flow enzyme immunoassay for the sensitive determination of skeletal troponin I (TnI) as a specific thermostable marker of muscle tissue [68,69] for the detection of pork additives in meat products [70]. The troponin test, owing to the specificity of mAbs against TnI clones IS7 and 6F9 (HyTest, Finland), differentiated the types of raw meat materials since mAbs only detected troponin in mammalian meat, not in birds. The same group of authors developed test systems to detect antibiotics in foods: aminoglycoside neomycin used in agriculture and veterinary medicine and presumably contaminated foodstuffs [71], and for the simultaneous determination of tylosin and lincomycin—antibiotics of the classes of macrolides and lincosamides that are also widely used in animal husbandry and implicated in food contamination [72,73].

Histamine is a biogenic amine that causes allergies and food poisoning, an important indicator of the freshness and quality of products. Monoclonal antibodies were obtained for this low-molecular compound, based on which the enzyme immunoassay and chemiluminescent enzyme immunoassay were developed. The methods demonstrated sensitivity, specificity, speed, and reliability in screening samples of foodstuffs and medicines. The developed immunoassays made it possible to detect trace amounts of histamine in food and drugs, the content of which was significantly lower than the maximum allowable content level established by various regulatory documents [98].

Staphylococcal enterotoxins cause food poisoning of varying severity. The long-term stability of toxins requires improvement to food quality control methods to prevent poisoning with staphylococcal enterotoxins. Based on mAbs, a method for specific and quantitative determination of staphylococcal enterotoxin E (SEH) in the format of a sandwich-enzyme immunoassay (linear range, 0.2–3 ng/mL) was developed [99]. A multiplex analysis was developed based on functional beads from BD Biosciences coated with monoclonal-trapping antibodies and biotinylated-monoclonal-detecting antibodies, as well as streptavidin conjugated with phycoerythrin for the simultaneous detection of three staphylococcal enterotoxins (SEA, SEB, and SEH) using flow cytometry. An effective application of the developed method for the analysis of food products was demonstrated [100].

### 6.3. Development and Application of Multiplex Test Systems Based on Analytical Microchips

Microchip technology is a fundamentally new level of laboratory research that allows the simultaneous testing of thousands of samples over a short time. An analytical microchip is an insoluble glass, plastic, gel, or silicon matrix with various biological objects and probes (antibodies, target proteins, DNAs, aptamers, or low-molecular-weight ligands) immobilized on it. Such microchips are intended to study the binding of molecules deposited on a substrate with partner molecules in a solution. Biochips in laboratory diagnostics are used in immunology and oncohematology to determine the risk of developing certain multifactorial diseases, individual sensitivity to certain drugs, etc. This direction is actively developing: Options for immobilizing specific probes on various types of matrices are being investigated, new reporter molecules are being created, and methods for registering signals are being improved.

In [101,102,103], examples of the use of microchips for medical purposes are described. The authors of [101] describe a multiplex analysis based on microchips on low-density hydrogels (hydrogel-based, low-density microarrays) for the simultaneous detection of autoantibodies against cytokines to diagnose polyglandular syndrome. Diagnosis of autoimmune endocrinopathies, including polyglandular syndrome, is difficult due to their rarity and non-specific clinical manifestations. Three-dimensional microchips for assaying hepatitis C in model systems and biological fluids were developed and successfully used in [102]. Antibodies against the surface viral protein E2 or CD81 fragments were used as detecting agents immobilized on the biochip. Polymer nanoparticles—analogs of the virus with E2 protein—were used to develop the method. The diagnostic value of the determination of specific antibodies in blood serum was demonstrated in [103]. The use of an immunochip with 12 recombinant antigens, markers of *Treponema pallidum* (Engelhardt Institute of Molecular Biology, Russian Academy of Sciences), made it possible to determine the content of specific immunoglobulins and determine the presence and stage of the disease.

In [104], a new strategy is proposed that solves the problems of non-specific binding and diffusion restrictions associated with the development of microchip-based assays. The essence of the method is to block the surface of the microchip with a blocking agent containing a perfluoroalkyl chain and a disulfide linker. The resulting surface becomes hydrophobic, and when the sample solution is cyclically drained and replenished, no immiscible liquid layer remains on it, ensuring an effective mass transfer of the analyzed substance to the micromatrix. Before the signal-detection procedure, disulfide bonds are chemically cleaved, and perfluoroalkyl chains are removed from the surface of the microchip along with non-specifically adsorbed proteins, which leads to an extremely low background. Using fluorescent detection, the authors showed a 30-fold increase in the signal-to-background ratio compared to a conventional glass substrate modified with epoxy resin. The combination of this method with the detection of magnetic beads led to a simple and hypersensitive immunoassay for cholera toxin, the detection limit of which was 1 fM. Effective mass transfer provided highly sensitive detection of whole viral particles, despite their low diffusion coefficient. The achieved limit of detection of the smallpox vaccine virus was 10^4^ per 1 mL of sample.

### 6.4. Detection of Diagnostic Antibodies 

This section discusses work devoted to the determination of diagnostic antibodies using various immunochemical methods.

An enzyme-immunoassay-based test system has been developed to detect antibodies against the infectious bursal disease virus (EBV), a disease affecting young chickens and harming poultry farming [105]. Natural human immunoglobulin M antibodies against Le^C^ have diagnostic value in breast cancer. The level of these antibodies was studied by the enzyme immunoassay method; their content in breast cancer patients was shown to be lower than in healthy women. The epitope specificity of these antibodies was characterized using a printed glycan array and enzyme immunoassay; the antibodies recognized a disaccharide but did not bind to glycans ending in Le^C^, which implies that it is impossible to bind to ordinary glycoproteins of non-malignant cells. The antibodies had a cytotoxic effect on breast cancer cells, which suggests that anti-Le^C^ plays a role in tumor surveillance [106].

A multiplex label-free biosensor has been designed to diagnose autoimmune diseases by the highly sensitive measurement of critical concentrations of autoantibodies in human serum, which correspond to their aggressiveness towards body tissues. The biosensor is based on the spectral-correlation interferometry and image processing of microarray glass biochips, which are microscope cover glasses without additional thin-film coatings available for single use. A highly sensitive detection and characterization of the activity of autoantibodies to thyroglobulin and thyroid peroxidase in the same serum sample after 25 min is described. The dynamic spectrum covered the entire range of clinically significant concentrations of autoantibodies. The developed method for characterizing the activity of autoantibodies by registering the kinetics of their binding to free native antigens is based on the polyvalence of autoantibodies. The proposed biosensor and the method of native kinetic registration can be used to develop new criteria for the complex diagnosis of autoimmune diseases, based not only on traditional concentration measurements but also on the quantitative assessment of the aggressiveness of autoantibodies [107,108].

In [109], a method for removing autoantibodies from the blood plasma of patients with autoimmune bullous dermatosis (pemphigus patients) is proposed. The authors used an approach based on creating a chimeric molecule combining an antibody Fc fragment and an EC2 desmoglein3 extracellular domain. The product was accumulated by transient expression in Chinese hamster ovary cells. To create an immunosorbent, a macroporous WorkBeads 40/10000 ACT matrix activated by bromohydrin-O-CH_2_CH(OH)-CH_2_Br groups was used. The use of this sorbent significantly reduced the concentration of immunoreactive immunoglobulins in plasma; their percentage decreased by 46%. At the same time, the immunosorbent was inert to the other blood plasma components.

Several publications are devoted not only to the detection of autoantibodies but also to their enzymatic properties. Thus, in [110], it was shown that IgG from the blood sera of patients with schizophrenia hydrolyzes myelin basic protein. Patients with a continuous type of schizophrenia have a more pronounced violation of myelination in the brain structures. This reflects the peculiarity of the immunological reactivity of the organism during this disease. Autoantibodies—abzymes—that hydrolyze microRNAs were found in the sera of patients with multiple sclerosis and systemic lupus erythematosus [111,112]. Evidence has been presented that the secretory IgA molecules of breast milk (but not the blood sera of healthy men and non-pregnant women) can effectively hydrolyze ribooligonucleotides, i.e., they are abzymes that effectively hydrolyze microRNAs in their loop and duplex regions. Feeding newborns with breast milk plays a very specific role and is a unique aspect of mammalian nutrition [113].

## 7. The Use of Physico-Chemical Methods for the Development of Immunosensors 

Early diagnosis of infectious diseases as soon as bacterial antigens and viruses is the most important task in developing a treatment strategy; the use of physico-chemical methods significantly increases the sensitivity of the determination. A range of work is devoted to this problem. 

**Figure 5:**
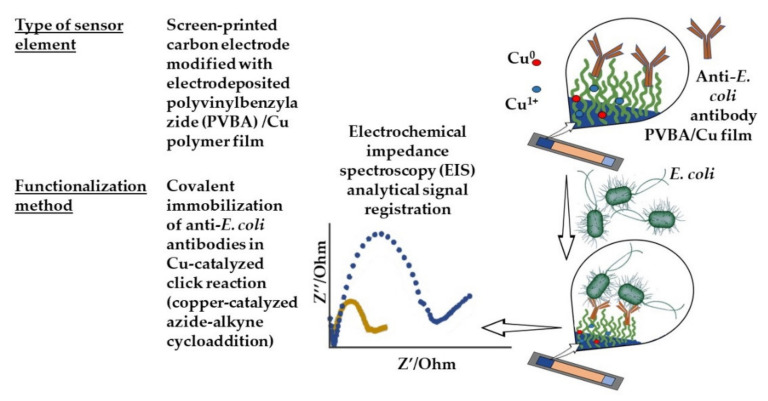
A label-free impedimetric immunosensor based on covalent antibody immobilization.

Sensors for voltammetry have been improved. An original method of covalent immobilization of anti-*E. coli* antibodies on the surface of screen-printed carbon electrode by the copper-catalyzed azide–alkyne cycloaddition reaction was developed [114] (Figure 5). A polyvinylbenzylazide film was applied to the electrode surface by the electrochemical method with the simultaneous inclusion of copper-containing particles in the structure. The modified electrodes were characterized by scanning electron microscopy, electrochemical impedance spectroscopy, and cyclic voltammetry. As a result of electrooxidation, Cu^+1^ ions were formed, which catalyzed the “click” reaction, which reduced the immobilization time of the immunoreceptor (antibodies) to 30 min. The developed label-free impedimetric immunosensor for *E. coli* detection demonstrated high stability and selectivity with respect to target bacteria. The detection limit was 6.3 CFU/mL, with a linear range of 10^3^–10^6^ CFU/mL.

**Figure 6:**
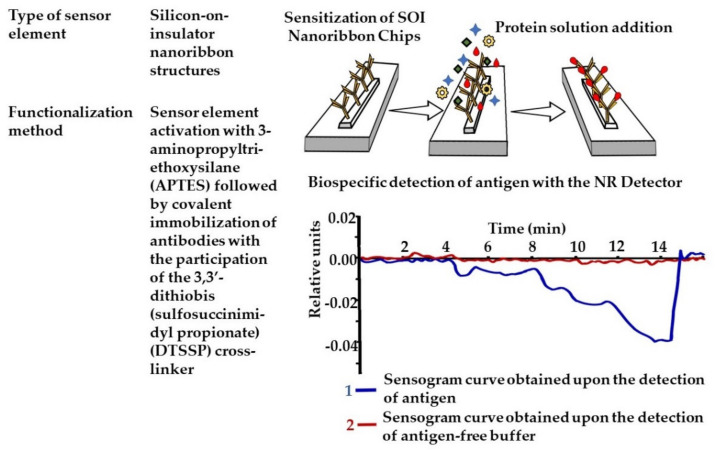
Label-free nanoribbon (NR) immunosensor.

The detection of influenza A virions using a nanoribbon detector has been demonstrated (Figure 6). The chips for the detector were fabricated using silicon-on-insulator nanoribbon structures (SOI nanoribbon chip) using a complementary metal-oxide-semiconductor compatible technology using gas-phase etching and standard optical photolithography. The nanoribbon detector is a molecular detector that allows individual biological macromolecules and viral particles to be identified while being counted, which determines the high speed and concentration sensitivity of the analysis. The operating principle of the detector is based on the registration of an electric current flowing through the detector. The surface of the chip with nanofilm contained a matrix of 10 sensor elements with nanoribbon. For the biospecific detection of anti-influenza virus A antibody, a catcher agent was covalently immobilized onto the sensor surface (and murine monoclonal antibodies against hepatitis B virus antigens, as control, obtained from the Federal State Budgetary Institution “State Research Center Immunology Institute,” Russian Federal Biomedical Agency, Moscow). The detection of the influenza A virus was performed by registering the source–drain current in real-time via the nanoribbon detector. Virus particles detected by antibodies changed chip conductivity. The lowest detectable concentration of viral target particles was 6 × 10^−16^ M (corresponding to 10^4^ viral particles/mL) [115].

**Figure 7:**
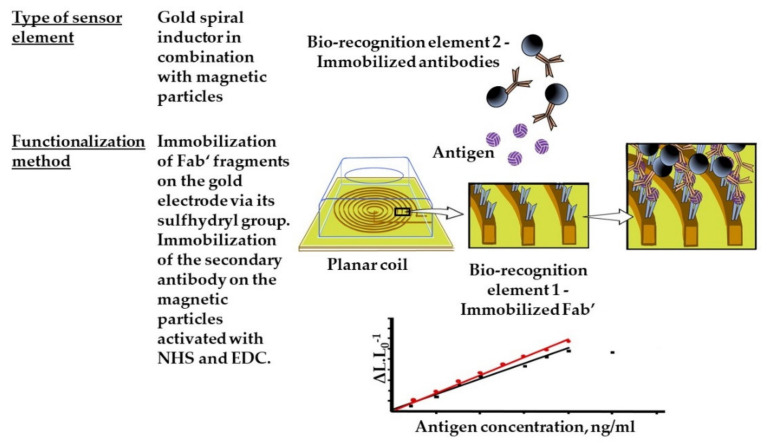
Magnetic inductive micro-electrode for virus monitoring.

Flat inductors are miniature biosensors that can be used as chips (Figure 7). A model for viral monitoring has been proposed for a magnetically inductive biosensor. Magnetic particles are attached to bioreceptors (antibodies) to form a sandwich on the surface of the inductors. The inductor includes a coil comprising 250 turns of a planar gold wire (thickness 200 nm, length 392 mm) placed in a circle of 2 mm diameter covered with a silicon oxide plate. The model was used as an immunosensor for the detection of hepatitis B surface antigens (HBsAg). The Fab-segment-recognizing HBsAg is immobilized on the coil. The second component of the immune sandwich is a polyclonal antibody (MyBioSource, USA) conjugated with magnetic particles. In the presence of HBsAg in the solution, the effect of magnetic particles on the coil’s inductance is recorded and used as a signal for detecting HBsAg. The magnetic-inductive immunosensor demonstrated specific responses to HBsAg with a detection limit of 1 ng/mL, and a linear range from 1 to 200 ng/mL [116].

As exemplified by the vector-borne transmissible gastroenteritis (TGE) virus, a method of detecting coronaviruses using an acoustic sensor was developed. The principle of electroacoustic analysis is the detection of fluctuations of a signal that results from specific biological antigen–antibody interactions, and the presence of extraneous viruses did not interfere with the determination. The presence of antiviral antibodies led to a change in the sensor parameters [117].

The problem of the widespread use of antibiotics and their methods of detection is very current today. The creation of a biosensor platform based on yttrium oxide (nY_2_O_3_) modified with chitosan (CH) has been described. The electrode was chemically conjugated with antibodies to fluoroquinolones (MyBiosource, USA). The developed biosensor was used to detect trace amounts of the antibiotic norfloxacin. The diagnostic characteristics of the fabricated sensor when measured using differential pulse voltammetry proved to be better compared to previously described biosensors and commercially available EIA in terms of sensitivity and a lower detection limit [118].

**Figure 8:**
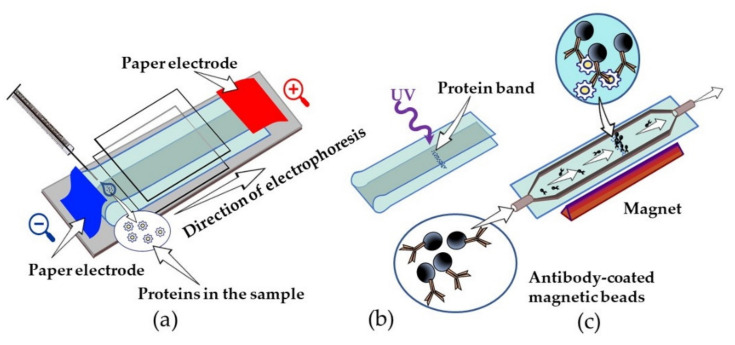
Rapid ultrasensitive gel-free immunoblotting with magnetic labels. (**a**) Separation of analyzed proteins on nitrocellulose under non-denaturing conditions; (**b**) photochemical immobilization of analyte; (**c**) antibody-coated magnetic bead visualization.

Some publications [119,120] have attracted attention for their non-standard solutions to the application of classical immunochemical methods. For example, they describe a new method of immunoblotting, characterized by an exceptional sensitivity, speed, and low uptake of antibodies. Instead of a polyacrylamide gel, a thin conductive layer between touching hydrophilic cellulose membranes separates the protein mixture (Figure 8). The separation of proteins is carried out under non-denaturing conditions, then the cellulose layer is smoothed by precipitation, and the photochemical immobilization of proteins is carried out in situ due to the preliminary modification of the azidophenyl groups’ cellulose. Thus, an additional stage of protein transfer from the gel to the membrane is excluded. To visualize the protein bands, the membranes are treated with magnetic beads coated with antibodies. Biotinylated monoclonal anti-human IL-1β antibodies (ThermoFisher, CRM57) and polyclonal anti-human IgA antibodies (ThermoFisher) were used. The unbound particles are removed using a magnet. It was possible to achieve unique detection limits for interleukin IL-1β, 0.3 fg, or ~104 molecules, despite the fact that the total blotting time was about 5 min. The method was also demonstrated to detect total IgA and IgA specific to the *Mycobacterium tuberculosis* antigens in exhaled air samples obtained from healthy subjects and patients with tuberculosis.

A method based on the homogeneous competitive immunoassay has been proposed; the analyte is detected by optical detection of a specific inhibition of the aggregation of nanoparticles that occurs when the analyte is present in the sample under study. Direct quantitative detection of small molecules (haptens) occurs using biosensing with dynamic light scattering; the nanosensor uses an effective concept of reducing the size of nanoparticle aggregates while increasing the analyte concentration due to competitive binding between analyte molecules in the solution and nanoparticles immobilized on the surface. It is this fact that enabled the application of this method to a low-molecular-weight analyte, not multi-epitope analytes, as previously. The method was tested in milk for the determination of chloramphenicol. This antibiotic, even in small concentrations, can cause serious side effects, so it is important for the diagnosis of food safety. High sensitivity was combined with a record-wide dynamic range (three orders of magnitude) of nanosensing [121].

**Figure 9:**
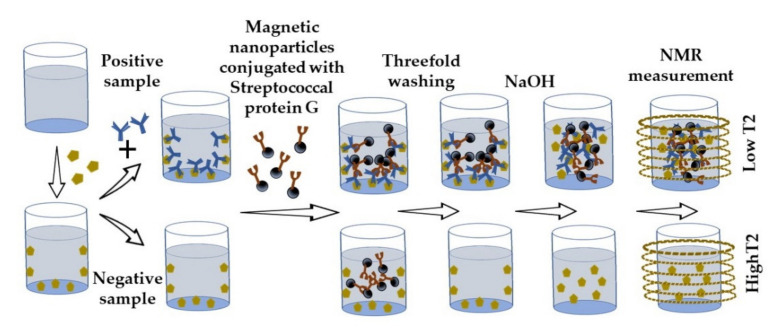
Nuclear magnetic resonance immunoassay based on the displacement of magnetic nanoparticles.

A nuclear magnetic resonance immunoassay has been developed based on carbon-coated iron nanoparticles conjugated with recognizing antibodies (Figure 9). The principle of the analysis is that EIA plates are covered with antibodies binding the analyte; then, the test solution is added, in which the content of the analyte is detected by the interaction of magnetic nanoparticles with detecting antibodies. mAbs (clones 3A6 and 1A6) manufactured by Bialexa (Russia) were used to detect the prostate-specific antigen. Next, magnetic nanoparticles were eluted from the surface of the wells into the solution. Separated magnetic nanoparticles reduced the values of the transverse relaxation time (T2) of protons from the surrounding solution. A portable nuclear magnetic resonance relaxometer was used to measure T2. The key novelty of the immunoassay is that the displacement of nanoparticles from a solid carrier by the eluting solution enables the advantages of solid-phase analysis to be combined with sensitive detection of changes in the transverse relaxation time in the liquid volume [122].

## 8. Conclusions

A large number of studies by Russian scientists published over the past two years and described in the review indicate intensive scientific work in the development and application of high-tech sensor systems based on antibodies and the high rates of development of Russian research thought. As shown in the review, there is practically no direction in the R&D of highly sensitive analytical systems that is not developed in Russia. The claim that antibodies are the key components of modern biosensors is widely confirmed in the work of Russian scientists.

## Figures and Tables

**Table 1:** Comparison of analyte-binding scaffolds in publications by Russian scientists.

Scaffold	Size, kDa	Original Protein	Structure	Source	Production System	Homogeneity	Affinity/Specificity
Polyclonal antibodies	150, 900	Full-size immunoglobulin	Globular proteins	Serum of immune animals	Animal	Heterogeneous with differing paratopes for an antigen	High affinity
Monoclonal antibodies	150	Full-size immunoglobulin	Globular proteins	Plasma cell of immune animals	Animal	Homogeneous with one paratope	High affinity for one epitope
scFv	28	Variable domain of IgG heavy and light chains	Predominant β-folding structure described for variable domain IgG	Combinatorial library-constructed-based B-cells	In both prokaryotic (mainly *E. coli*) and eukaryotic (yeast, insect cells, plant cells, and mammalian cells) systems	Homogeneous	Micromolar–nanomolar after first rounds of biopanning
VhH	12–15	Variable domain of heavy chain of camelid antibody	Folded β-sheet	Combinatorial library-based B-cells of Camelidae	In both eukaryotic and prokaryotic systems (*E. coli*, *Saccharomyces cerevisiae*)	Homogeneous	High target affinity and specificity
DARPins	14–18	Natural ankyrin repeats	α-helical + β-turn	Combinatorial library	*E. coli* (up to 200 mg/L)	Homogeneous	Nanomolar–picomolar affinity
Affibodies	6	Z domain of staphylococcal protein A	α-helical	Combinatorial library	Peptide synthesis and *E. coli*	Homogeneous	Nanomolar–picomolar affinity
ABD	5	Albumin-binding domain of streptococcal protein G	α-helical	Combinatorial library	Peptide synthesis and *E. coli*	Homogeneous	Nanomolar–picomolar affinity

**Table 2:** Practical application features of analyte-binding scaffolds.

Scaffold	Preferred Application	Advantages	Limitations
Polyclonal antibodies	For detecting proteins with low content; as secondary antibodies in immunoassays; as capture antibodies in sandwich EIA; detection in solutions with different pH and salt concentrations; immunoprecipitation	Cost-effective production compared to mAbs, about three months; easily labeled without loss of binding capacity; resistant to minor changes in antigens	High-probability immune cross-reactivity; contain non-target antibodies; background noise; variability of lots
Monoclonal antibodies	For quantitative analyses; for staining cells with less background	Stable preparations; production of highly concentrated quantities possible	Costly production, about 6 months; necessary gene engineering (humanization) to reduce immunogenicity
scFv	As a capture and/or detection agent in immunosensors and immunoassays: colorimetric (EIA); fluorescence (including FRET); chemiluminescense; luminescense; immuno-PCR; immunoelectron microscopy; electrochemical; quartz-crystal microbalance; SPR; piezoelectric microcantilever; simultaneous recognition of multiple epitopes; using bi- and tri-specific constructs; detection of toxins and venoms	Cost-effective production compared to mAbs; time-consuming procedure selection from combinatorial library (about 2 weeks for primary selection); fast tissue penetration; the possibility of genetically engineered labeling	Lack of effector functions; reduced thermal stability compared to mAbs
VhH	As a capture and/or detection agent in immunoassays: radiochemical; crystallography; ELISA; SPR; piezoelectric microcantilever; predominantly for cancer target recognition, bioimaging; drug delivery; detection and neutralization of toxins and venoms; definition of autoantigens	Cost-effective production compared to mAbs; time-consuming procedure selection from combinatorial library (about 1 month for primary selection); highly soluble; thermally and pH stable; fast tissue penetration; special conformational diversity; recognize epitopes, which are not immunogenic for conventional mAbs; low immunogenicity; the possibility of genetically engineered labeling	Lack of effector functions
DARPins	Predominantly for cancer target recognition, bioimaging; drug delivery	Cost-effective production compared to mAbs; time-consuming procedure selection from combinatorial library (about 1 month for primary selection); high thermal and pH stability; fast tissue penetration; the possibility of genetically engineered labeling	Lack of effector functions; required to increase the serum half-life by increasing their molecular size
Affibodies	Predominantly for cancer target recognition, particulary by electrochemical impedance spectroscopy; bioimaging; drug delivery; detection of toxins	Cost-effective production compared to mAbs; time-consuming procedure selection from combinatorial library (about 1 month for primary selection); high thermal and pH stability; fast tissue penetration; the possibility of genetically engineered labeling	Lack of effector functions; required to increase the serum half-life by increasing their molecular size; immunogenicity of protein A
ABD	Cancer target recognition, particulary by immuno-positron emission tomography; bioimaging; drug delivery	Cost-effective production compared to mAbs; time-consuming procedure selection from combinatorial library (about 1 month for primary selection); high thermal and pH stability; fast tissue penetration; the possibility of genetically engineered labeling	Lack of effector functions; immunogenicity of protein G

## Data Availability

Not applicable.
